# A common data model for the standardization of intensive care unit medication features

**DOI:** 10.1093/jamiaopen/ooae033

**Published:** 2024-05-02

**Authors:** Andrea Sikora, Kelli Keats, David J Murphy, John W Devlin, Susan E Smith, Brian Murray, Mitchell S Buckley, Sandra Rowe, Lindsey Coppiano, Rishikesan Kamaleswaran

**Affiliations:** Department of Clinical and Administrative Pharmacy, University of Georgia College of Pharmacy, Augusta, GA 30912, United States; Department of Pharmacy, Augusta University Medical Center, Augusta, GA 30912, United States; Division of Pulmonary, Allergy, Critical Care and Sleep Medicine, Emory University, Atlanta, GA 30322, United States; Northeastern University School of Pharmacy, Boston, MA 02115, United States; Division of Pulmonary and Critical Care Medicine, Brigham and Women’s Hospital, Boston, MA 02115, United States; Department of Clinical and Administrative Pharmacy, University of Georgia College of Pharmacy, Athens, GA 30601, United States; Department of Pharmacy, University of North Carolina Medical Center, Chapel Hill, NC 27514, United States; Department of Pharmacy, Banner University Medical Center Phoenix, Phoenix, AZ 85032, United States; Department of Pharmacy, Oregon Health and Science University, Portland, OR 97239, United States; Department of Biomedical Informatics, Emory University School of Medicine, Atlanta, GA 30322, United States; Department of Biomedical Engineering, Georgia Institute of Technology, Atlanta, GA 30322, United States; Department of Biomedical Informatics, Emory University School of Medicine, Atlanta, GA 30322, United States; Department of Biomedical Engineering, Georgia Institute of Technology, Atlanta, GA 30322, United States

**Keywords:** artificial intelligence, common data model, critical care

## Abstract

**Objective:**

Common data models provide a standard means of describing data for artificial intelligence (AI) applications, but this process has never been undertaken for medications used in the intensive care unit (ICU). We sought to develop a common data model (CDM) for ICU medications to standardize the medication features needed to support future ICU AI efforts.

**Materials and Methods:**

A 9-member, multi-professional team of ICU clinicians and AI experts conducted a 5-round modified Delphi process employing conference calls, web-based communication, and electronic surveys to define the most important medication features for AI efforts. Candidate ICU medication features were generated through group discussion and then independently scored by each team member based on relevance to ICU clinical decision-making and feasibility for collection and coding. A key consideration was to ensure the final ontology both distinguished unique medications and met Findable, Accessible, Interoperable, and Reusable (FAIR) guiding principles.

**Results:**

Using a list of 889 ICU medications, the team initially generated 106 different medication features, and 71 were ranked as being core features for the CDM. Through this process, 106 medication features were assigned to 2 key feature domains: drug product-related (n = 43) and clinical practice-related (n = 63). Each feature included a standardized definition and suggested response values housed in the electronic data library. This CDM for ICU medications is available online.

**Conclusion:**

The CDM for ICU medications represents an important first step for the research community focused on exploring how AI can improve patient outcomes and will require ongoing engagement and refinement.

## Introduction

Information technology (IT) is a rapidly growing field of investigation in critical care.^1–8^ For computers to correctly interpret data generated by critically ill patients, precise feature selection and standardized, machine-readable ontologies are necessary to create robust, well-validated prediction models and clinical decision support systems (CDSS).^9^^,^^10^ A significant challenge facing the informatics community is creating these standardized ontologies for the different components of electronic health record (EHR) (eg, laboratory values, vital signs, clinical interventions, etc). For example, when clinical data are pulled from data warehouses regarding serum bicarbonate values, these may read as “serum bicarbonate,” “bicarbonate,” “CO_2_,” “HCO_3_,” or any number of institution-specific monikers. Without standardization, IT tools may interpret each of these values differently, which can be prohibitive for validating algorithms in external datasets. With the increasing interest in artificial intelligence (AI) models, these issues have become compounded.

Standardization that ensures each laboratory test result (or clinical intervention) will always be referred to by the same name (and ideally the same unit of value or exposure) requires a common data model (CDM) to facilitate both comparisons among datasets and adaptation and improvement of algorithms and models among different investigator teams. A CDM refers to any data model generally consisting of an ontology and associated metadata that allows for standardized data and information exchange among different applications and data sources. CDMs facilitate reproducibility and generalizability across datasets, and without these efforts, the external validity of the model or tool is jeopardized.^11^ The importance of these CDM development efforts has been internationally recognized with the publication of the FAIR Guiding Principles, which are intended to steward scientific data to be Findable, Accessible, Interoperable, and Reusable.^12^ The Observational Medical Outcomes Partnership (OMOP) is a leading CDM for clinical data and has undertaken structuring health data with the goal to connect previously disparate datasets.^13^ However, OMOP has taken only preliminary steps towards incorporation of complete medication data, particularly as it relates to the complexity of medication use inherent in the intensive care unit (ICU), with those steps mostly focusing on drug product information (eg, name, formulation).

Complex medication regimens are commonly used when treating critically ill patients. The medications in these regimens independently affect both treatment and safety outcomes.^14–16^ Beyond formulation data, underlying patient features affect drug dosing, drug response (including efficacy and safety), and patient outcomes. These features have not been considered in current CDMs, but without these data, IT tools may view different medication orders the same way despite big differences, including indication for use or pharmacokinetic/pharmacodynamic response. For example, the same heparin product may be given for the routine prophylaxis of venous thromboembolism *and* the management of a life-threatening pulmonary embolism. In this case, the indication and expected patient response are highly disparate. This computer misinterpretation has ramifications from appropriate reporting in demographics tables of clinical studies to inappropriate AI-based predictions or CDSS.

Thus, without a rigorously enhanced CDM for ICU medications, the ability to use IT tools to support medication therapy optimization remains in its infancy. However, the development of such a CDM for all 20 000 Food and Drug Administration (FDA)-approved drug products that includes clinically meaningful features while still being able to interface with existing patient data requires substantial resources and clinical validation. In this paper, we sought to develop the groundwork for CDM for ICU medications (ICURx) to standardize the medication features needed to support future ICU IT applications with the goal to identify necessary medication features and provide an initial library of relevant ICU medications.

## Materials and methods

### Design

A consensus process based on a modified Delphi approach was initiated in April 2022. This method has been successfully applied in healthcare research efforts including common data element development, prescribing guideline generation, and medication outcome prioritization.^17–21^ Choosing this approach allowed the team to address temporal and cost constraints while ensuring each individual subject matter expert had an equal voice.^22^

### Participants

The 9-member expert panel was invited to participate in a 5-round modified Delphi process, including 1 computer scientist, 1 intensivist, and 7 critical care pharmacists. All 8 clinicians represented geographically diverse locations and held critical care board-certification in their respective professions; therefore, each were deemed content experts in the domain of critical care pharmacotherapy and able to cast votes regarding the clinical significance all CDM attributes. The computer scientist contributed to framing the process and providing important insights for CDMs but deferred on clinical considerations. The University of Georgia Institutional Review Board (IRB) deemed this project to be exempt from IRB review (PROJECT00006204). Voluntary involvement in the Delphi process implied participant consent.

### Framework

Participants were briefed on the primary goal of the expert panel: to develop a CDM for ICU medications for use in AI applications. A framework consisting of concept, purpose, and ideal characteristics for this CDM was supplied to the panel to facilitate a consistent development process (**see**Table 1). *A priori*, it was defined each CDM would be comprised of a list of medication features and standardized definitions and coded suggestions. In machine learning, a feature can be thought of as an individual, measurable property or characteristic of a particular entity. For any given medication, an individual feature could include dose, route, the risk for drug–drug interactions, etc.^23^ The group was asked to consider various situations focused on how ICU clinicians delineate unique features among different medications used in the ICU (see Table 2).

**Table 1.** Framework for development of the common data model for ICU medications.

**Concept** • For use in artificial intelligence and other data science applications investigating critical illness **Purpose** • Provide an intensive care unit medication ontology that meets the Findable, Accessible, Interoperable, and Reusable (FAIR) Guiding Principles **Characteristics** • Ability to distinguish unique medication identities and profiles • Captures relevant medication features used in clinical decision-making • Feasible to incorporate into more definitive database

**Table 2.** Proposed cases for developing machine-readable ICU medication features.

**Scenario 1.** *Aspirin 81 mg vs acetaminophen 325 mg*
While a machine will tend to see continuous numbers as ranking above or below each other, clinicians view these numbers as ordinal values, with each gradation being associated with unique profiles of efficacy and safety (eg, starting dose, maximum dose, etc). Without appropriate coding, the machine can interpret 325 mg as “greater than” 81 mg, which while numerically true, is not the same way a clinician would interpret this medication, which is that both are being given at low or starting doses. Ironically, 325 mg of acetaminophen is often considered a “low dose” while 325 mg of aspirin would be considered an inappropriately high dose in most cardiac indications.
**Scenario 2.** *Intravenous bolus midazolam vs continuous infusion midazolam* The same medication given by the same route can be considered unique by a clinician (due to significantly different pharmacokinetic profiles). Intravenous midazolam given as a one-time bolus is known for its “quick on, quick off” effect and is a first-line agent for procedural sedation. In contrast, midazolam as a continuous infusion has a profoundly different pharmacokinetic profile, notable for its long duration of effect due to drug accumulation that can result in increased duration of mechanical ventilation, delirium, and other adverse effects. Without appropriate coding, these 2 forms of intravenous midazolam could be considered interchangeable by a computerized algorithm.
**Scenario 3.** *Cefepime 1 g every 24 hours vs Cefepime 2 g every 8 hours.* The team was asked to consider maximal dosing. For example, cefepime is often dosed at 2 g every 12 hours for a critically ill patient with normal renal function, except in renal failure when the dose is 1 g every 24 hours. However, in renal failure being treated with certain forms of continuous renal replacement therapy, the dose may be 2 g every 8 hours. As such, a patient that received 1 g every 24 hours may be receiving the appropriate dose or a 4- to 6-fold underdose depending on the circumstances.

### Study sample

A representative ICU medication list was derived from the unified electronic health record (EHR) from a random sample of 1000 ICU patients at the University of North Carolina Health System between October 2015 and October 2020. Inclusion criteria for patients consisted of age ≥18 years old on their index ICU admission who had been admitted to the burn, cardiac, cardiothoracic surgery, medical, mixed, neurosciences, or surgical ICU. Exclusion criteria included patients with active comfort care orders.

Given the Federal FDA has approved over 20 000 *medication products* and that each individual medication product can be ordered in a myriad of unique ways, a nearly infinite number of *medication orders* exists. For example, the medication product of “aspirin 325 mg tablet” may be ordered as once daily, 3 times daily, 3 times daily as needed for headache, etc. To improve the feasibility of this initial process, the team focused on 2 key areas: (1) medication products (eg, aspirin 325 mg tablet) and (2) medication products deemed highly relevant for ICU patients as identified using a previously validated metric (the Medication Regimen Intensity—Intensive Care Unit [MRC-ICU] score).^14^^,^^15^^,^^24–31^ Given medications in the MRC-ICU have been previously identified as common to ICU care and having unique characteristics that associate their use with increased ICU patient care complexity and a requirement for careful monitoring, they were deemed to be a relevant initial list of representative medications for CDM development.^14^^,^^15^^,^^24–31^ Structuring a CDM to interpret patient-level medication orders was deemed beyond the scope of this effort, although it is an important area for future work. The methodological decisions to focus on a select list of medications and 2 primary domains of medication features were made in deference to the overall scope of our project, given that each one of the 20 000 medications can be ordered in a multitude of ways. Indeed, over 30 000 unique orders were placed within this dataset alone.

### Modified Delphi process

**Figure 1.**
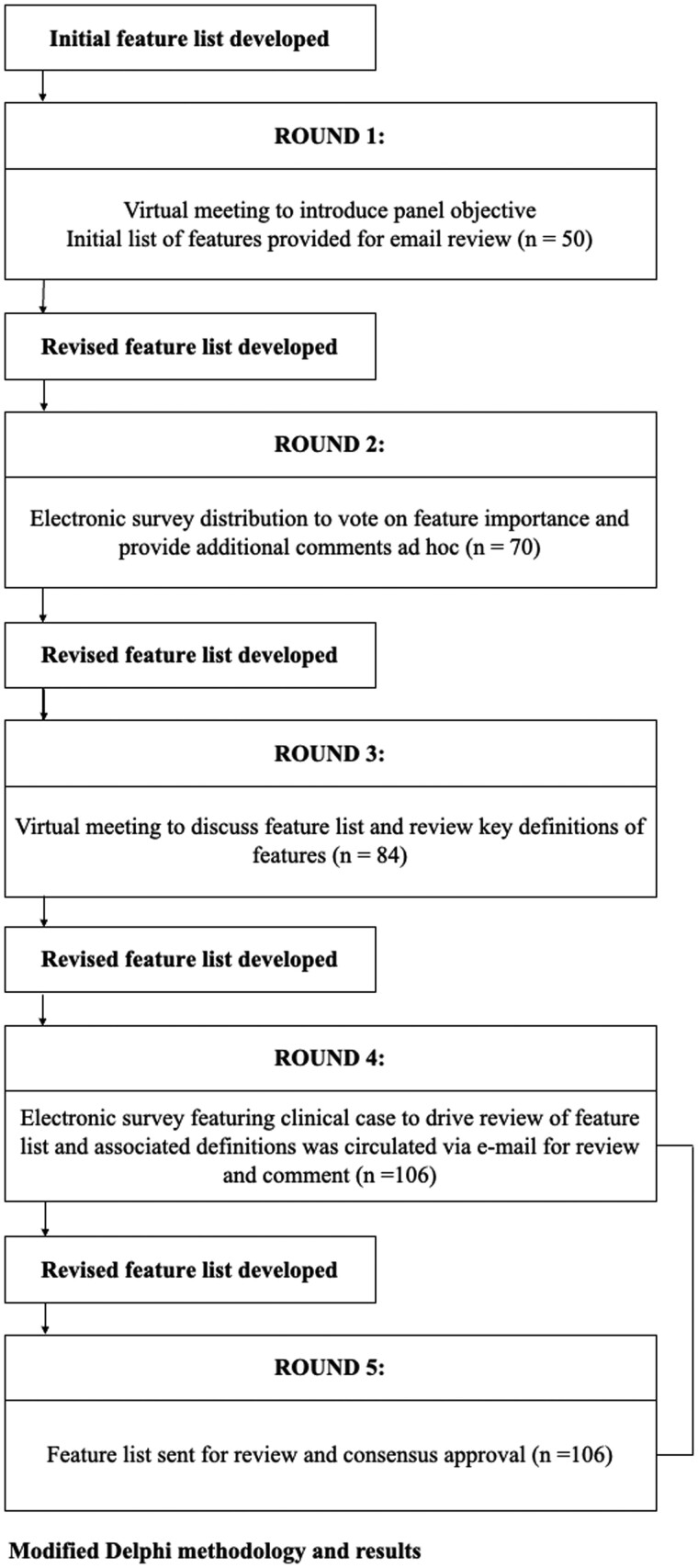
Modified Delphi process for development of the common data model for ICU medications.

The modified Delphi process is depicted in Figure 1. Prior to the virtual meeting for Round 1, a description of the objective and an initial list of 50 potential medication features were electronically sent to each participant to provide a starting point for independent brainstorming. In Round 1, the goal was to compile and define an initial series of machine-readable features (or common data elements) that characterize medications and influence ICU clinical decision-making. Each member of the panel was provided the opportunity to independently review an initial list of features, provide feedback, and suggest additional features. All Round 1 feedback from the expert panel was evaluated and incorporated into a revised library for second round deliberation.

In Round 2, the goal was additional item generation. An electronic survey was administered regarding feature importance (see Table S1). A survey, intended to be completed independently and anonymously, was provided to each panel member to vote on the relative importance of each feature in the library (see Table S1). Comments about new features and feedback on existing features was also encouraged. The results of this survey were incorporated into a new list of common data elements and distributed to each member.

In Round 3 of the Delphi process, the expert panel met to review and discuss the results of Rounds 1 and 2, including each of the new features added. The goal of this round was to identify broad themes of the common data elements generated and prioritize which elements should be included in the first CDM version.

Following this in-group discussion, Round 4 consisted of review and comment regarding an updated list of medication features. In this round, a patient case was presented that included a medication regimen. The prompts asked the panel to consider features “that would be most relevant and most important to clinical decision-making associated with comprehensive medication management of that patient” and vote for inclusion into 3 categories: (1) a *core list* of features for use in ICU AI algorithms not specifically focused on medical use; (2) a *core list* of features for use in ICU AI algorithms targeted to medication use optimization; and (3) an *expanded list* of features for use in ICU AI algorithms targeted to medication use optimization. While feasibility and parsimony were considered, they were deemed secondary priorities given the general construct of AI is to maximize the use of available data and the ultimate goal of this library was for it to be able to be readily linked to any investigator’s dataset. Inclusion in the first category was defined as having greater than or equal to 4 votes (see Table S2).

In Round 5, a final list of features and their definitions was sent out for approval that the panel believed adequately represented key features of medications used in the ICU. Approval was indicated by anonymous voting. The list of core features is provided in Table 3. The complete list of features and their associated definitions is provided in Table S3. Entries were created for all features for the list of medications. To reduce discrepancies among sources, the primary data source used was Lexicomp Online (Waltham, MA: UpToDate, Inc), which was chosen for its completeness and relevance to the ICU.^32^^,^^33^ Certain clinical features (eg, if a different route represented a potential escalation in care) were coded based on the expert opinion of a clinician panel board certified in critical care pharmacotherapy.

**Table 3.** List of medication features.

Drug product features	
Drug product (core) Drug name (core) Therapeutic category (core) Pharmacologic class (core) Formulary key drug type (expanded) Formulation strength (core) Formulation strength conversion (expanded) Converted drug name (core)	Converted drug strength (core) Strength units (core) Route (core) Intravenous volume (expanded) Concentration (expanded) Intravenous, sub-category (core) Oral, sub-category (core)

**Clinical features**	

**Dosing information**	**Laboratory monitoring**

Fixed route (expanded) Continuous infusion starting rate (core) Continuous infusion maximum rate (core) Units for continuous infusion (core) Weight-based dosing (core) Maximum daily dose available (core) Maximum daily dose value (core) Maximum daily strength units (core) Maximum daily dose for creatine clearance < 10 mL/min or dialysis (core) Maximum daily dose for creatine clearance = 10–29 mL/min (core) Maximum daily dose for creatine clearance = 30–49 mL/min (core) Maximum daily dose for creatine clearance < 10 mL/min or dialysis and critically ill (expanded) Maximum daily dose for creatine clearance = 10–29 mL/min and critically ill (expanded) Maximum daily dose for creatine clearance = 30–49 mL/min and critically ill (expanded) Maximum daily dose for creatine clearance < 10 mL/min or dialysis and has cystic fibrosis (expanded) Maximum daily dose for creatine clearance = 10–29 mL/min and has cystic fibrosis (expanded) Maximum daily dose for creatine clearance = 30–49 mL/min and has cystic fibrosis (expanded) Renal dose adjustment (core) Hepatic dose adjustment (core) Contraindicated in renal failure (core) Contraindicated in hepatic failure (core) Role in prophylaxis (expanded) Maximum prophylactic dose (expanded)	Sodium (core) Potassium (core) Serum creatinine (core) Glucose (core) Aspartate aminotransferase (core) Alanine transaminase (core) Magnesium (core) Creatinine kinase (core) Albumin (core) Triglycerides, lipid panel (core) Activated partial thromboplastin time (core) International normalized ratio (INR) (core) Anti-Xa (core) Hemoglobin (core) Hematocrit (core) Platelets (core) Absolute neutrophil count (core) White blood cell count (core) Microbiology results (cultures, sensitivity, rapid results) (core) Phosphorous (expanded) Serum osmolality (expanded) Ammonia (expanded)

**Vital sign and other ICU monitoring**	**Clinical decision-making**

Mean arterial pressure (core) Systolic blood pressure (core) Diastolic blood pressure (core) Heart rate (core) Respiratory rate (core) Temperature (core) Creatinine Clearance (core) Urine output (core) Dialysis (core) Patient weight (core) QTc interval (core) Bowel movements (core) Train of four (core) Pain scale (core) Sedation scale (core) Delirium (CAM-ICU) (core)	MRC-ICU presence (core) MRC-ICU weight (core) Route escalation (core) Patient acuity (core) Critical illness based dosing (core) ISMP high alert status (core) ISMP confused drug names (core) Therapeutic drug monitoring (core) Parameter based dosing (core) CYP3A4, 2D6, 1A2, 2C9, 2C19, P-glycoprotein Interaction (core) Drug indicates critical illness (core) Adverse events (expanded) Realm of the unusual (expanded) Beers Criteria (expanded) Beers Criteria Strong Recommendation (expanded) Pharmacogenomics (expanded) Broad vs narrow spectrum (expanded) Vancomycin-resistant *Enterococcus spp.* (expanded) Methicillin-resistant *Staphylococcus aureus* (expanded) *Pseudomonas aeruginosa* (expanded) Extended spectrum beta lactamase (expanded) Treatment failure (expanded)

The data underlying this article are available in GitHub (https://github.com/sikora07/ICURx), under ICURx at: https://www.icurxforai.com/.

## Results

### Study sample

From the random sample of 1000 patients, 9 patients were excluded given the ICU admission did not represent their index ICU admission. The derived medication list was comprised of 30 550 discrete medication order entries for 991 ICU patients.^24^ When filters for generic drug name, dose, and administration route were applied, a total of 1868 unique medication products were identified. When only those medications incorporated in the MRC-ICU Scoring Tool were considered, a total of 889 discrete medication products remained for review and coding by the panel.

### Modified Delphi process

As a result of Round 1 and the subsequent electronic discussion, the medication feature list expanded from 50 to 70. After the end of the virtual meeting for Round 3, a total of 84 features (along with definitions) were generated. Upon completion of the case in Round 4, features were expanded to a total of 106 (see Table S2). Following voting, a total of 71 core features were identified (see Table 3). The final CDM approved by the panel can be found in Table S3.

Our process revealed 3 distinct feature domains: (1) *Drug product features:* nature of the product used including drug name, drug dose, formulation, and route of administration; (2) *Clinical features*: medication characteristics driving ICU clinical decision-making regarding drug product use (eg, the risk for a known adverse drug event in the setting of critical illness); and (3) *Order features*: specific way in which the drug product was ordered for the patient (ie, the “sig”) (eg, acetaminophen 325 mg oral tablets could be ordered on a scheduled basis as 2 tablets every 6 hours or on a “prn” basis as 2 tablets every 4 hours as needed for a pain score ≥ 4/10). Given the construction of this medication product list which did not include medication orders and their associated features, only the Drug Product Feature and Clinical Feature domains were further developed; however, this area was noted to be an important area of future research as clinicians may view medications differently when viewed “in a silo” versus in context of the entire medication profile or the specific order. Within the clinical feature domain, 4 medication product-related feature categories were identified: dosing, laboratory monitoring, clinical monitoring (including vital signs), and clinical decision-making.

### Common data model

The CDM for ICU medications is housed electronically (icurxforai.com). It consists of 94 234 coded elements for 889 medications and 106 medication features. A data dictionary defines and expands upon the various features and provides standardized coding.

## Discussion

This paper describes the development of the first CDM focused on medications used in the ICU and includes a medication library of 889 ICU medications described by 106 individual features. Our efforts represent the first medication CDM development effort to prioritize clinical features through an expert, modified Delphi process and represents an important next step for IT in the ICU, which has significant potential to improve patient outcomes via both AI-based prediction of ICU complications as well as via CDSS.

Medications represent an important domain for critical care predictive algorithms and CDSS because they serve as both treatments to life-threatening illness but also independent risk factors for life-threatening complications due to adverse drug events (ADEs).^14^^,^^34^^,^^35^ Medications in the ICU setting are uniquely challenging to manage because many are high risk (ie, beneficial effects are also associated with important potential safety concerns) and may have a narrow-therapeutic index (ie, a narrow difference in the medication dose resulting in benefit vs side effects). The fact that medications in the ICU setting can be used in a nearly infinite number of combinations often results in the creation of complex, multi-drug medication regimens necessitating optimization by critical care clinician experts. Key ICU medication clinical decision-making components include drugs with synergistic mechanisms, overlapping side effect profiles resulting in ADE risks that are additive, and the fluctuating nature of critical illness resulting in frequent pharmacokinetic and pharmacodynamic alterations.^36^ While ICU clinicians routinely apply their knowledge of ICU medication features to manage the benefits and harms of medication therapy, this process has no equivalent in the AI domain given its unstructured and complex nature that precludes the creation of robust machine-readable libraries.^36^ Our team has previously shown that incorporating medication data into prediction models (both in the context of machine learning and traditional regression) can improve both prediction modeling and unsupervised analyses.^28^^,^^37–43^

Despite the importance of medications on outcomes, only simplistic, non-ICU-based CDMs (limited to features like medication name, dose, and route) have been developed.^9^^,^^36^^,^^44^ Current medication CDM efforts including the RxNorm by the National Library of Medicine^45^ and the Observational Medical Outcomes Partnership (OMOP)^13^ CDM have prioritized reproducibility when organizing medication products and have not yet considered many of the more clinical aspects of medication prescribing and outcomes. The primary goal of RxNorm is to normalize drug products using identifying codes (eg, “Albuterol 0.417 MG/ML Inhalation Solution” coded 351136).^46^ Realizing the FDA has approved more than 20 000 drug products the efforts of RxNorm are commendable; however, the provision of relevant features needed for clinical decision-making represents a key next step in this infrastructure that will further support CDSS development. Examples of present limitations with OMOP include the inability to characterize infusion medications (eg, titrations, total dose delivered, infuse rates) or the complexity of administration routes even within larger categories like “intravenous” (eg, piggyback, titration, push, bolus, etc) that have clinical as well as operational implications. Moreover, drug products are not connected to order or clinical information. To illustrate these issues, several examples are provided.

1) To simplify the complexity of ICU medication regimens received, an investigator might choose to place haloperidol, propofol, cisatracurium, levetiracetam, and hydromorphone in a “neurology” category. While pharmacologically true, these medications work in various capacities in the nervous system: the indication, safety profile, and ramifications are different, ranging from seizure prophylaxis to status epilepticus, procedural sedation for a broken bone to supporting extracorporeal membrane oxygenation. Neuromuscular blockers like cisatracurium work in the peripheral rather than central nervous system. Unique interactions between individual drugs are entirely lost with such a schema, and appropriate interpretation of how medications impact outcomes varies widely. Midazolam may be used for prolonged sedation vs. brief procedural sedation, with the former associated with significant adverse outcomes and the latter considered a drug of choice, in each case because of its mechanism of action and pharmacokinetic profile. Investigators run the risk of losing highly meaningful information relevant to understanding the effect of their treatment upon the outcome of interest. Next-generation CDS capable of interpreting medication dosing in the context of patient-specific features (not just a generally acceptable dosing range) and efforts to refine clinical pop-up alerts require this appropriate CDM infrastructure.
2) Norepinephrine, available in different salt formulations that each deliver different doses of the parent drug, represents a recent example of how glossing over specific medication details can impact research including clinical trials, pooled estimates in meta-analyses, and application of research findings to bedside care.
3) It is common practice to read a study that states “appropriate antibiotics” were started for sepsis (but without knowledge of the drug, dose, frequency, or indication) or management of refractory shock was “assessed” but without report exactly which pressors and infusion rates were used.
4) Without the dose, alteplase could be for a life-threatening pulmonary embolism or for routine maintenance of intravenous access.

The “devil is in the details” when it comes to interpreting medication data.^47^ The establishment and use of a CDM for ICU medications has the potential to unearth and resolve these issues, of which the critical care community is only presently becoming aware of.^47–49^ Acknowledging the complexity of ICU comprehensive medication management optimization, our effort sought to capture the nuances of clinical decision-making and incorporate it into a CDM through a multiple-step, consensus-based process involving an interprofessional group of experienced ICU clinicians and an AI methodologist. Our Delphi process allowed for nuanced discussion of the ICU medication clinical decision-making process not captured in the package insert or other standardized drug references. A notable strength of this process included grounding CDM development discussions in patient case scenarios. This process resulted in all medication features coded and defined as a CDM that extends beyond drug product mappings of the present-day CDMs to the realm of clinical decision-making for use in ICU AI applications. Incorporation of ICU medications into AI algorithms is in the early stages of development. Algorithms have been previously developed that include indicators of a broad class of medications, such as use of inotropes and vasopressors, without adjusting for the individual’s overall regimen.^37^^,^^50–56^ While such broad strokes were often used due to limitations of the technology, large amounts of relevant detail were ignored, given the unique pharmacokinetic and pharmacodynamic profile of each drug and potential interactions with other drugs that go unaccounted for with that approach. Unsupervised machine learning using Restricted Boltzmann Machine has been applied to the medication administration records of ICU patients revealing unique pharmacophenotypes associated with patient outcomes. The first iteration used just generic drug name in addition to basic patient demographics and ICU outcomes, excluding all other potential drug features, but still showed the presence of pharmacophenotypes.^24^ The second employed a significantly expanded list of drug features in line with this proposed CDM, showing again the ability of AI to incorporate vast amounts of data into its predictive algorithms.^37^ Understanding how to translate these pharmacophenotypes into clinically meaningful subgroups or interventions for a bedside clinician remains an ongoing area of investigation. Medication data have also been incorporated into supervised learning for prediction of prolonged duration of mechanical ventilation and mortality; however, neither of these analyses included the entire ICU medication regimen and focused just on generic name.^28^^,^^50–52^ Overall, a common theme is the improvement of modeling performance with the inclusion of medication data (particularly drug name or class), though little has been done incorporating comprehensive medication data for the patient’s regimen (including both all medications they received and relevant information on those medications).

Despite the strengths of our process, several potential limitations exist. Our process did not include all medications that can be used in the ICU, which will be an important area of future development given both steroids and diuretics were excluded from the original MRC-ICU score and patients undergoing end of life care were excluded. Second, the profile for each medication feature may not always be complete (eg, possible non-cytochrome P450 drug–drug interactions that could influence medication response) and while development of such features was considered out of scope for this initial process, additions are likely warranted. Although great care was taken to ensure proper coding, some of the CDM features are based on the expert opinion of our panel, which may differ from the viewpoints of ICU clinicians who were not involved. Discrepancies among data sources exist; how this should be best managed requires further research.^57^ Feature selection was based on those elements felt to be most germane to how each medication influenced clinical decision-making and comprehensive medication management. However, given the inherent differences among medications, this decision resulted in “not applicable” designations for some medications (eg, IV medication characteristics are not applicable to an oral formulation). Future research focused on defining the medication features most important to parsimonious model development that may include feature ablation is important.

We consider our proposed CDM for ICU medications to be a living and evolving database: iterative additions and revisions are required to continue to add available knowledge (eg, pharmacokinetic parameters, vesicant risk, suspension type; eg, the propylene glycol used to formulate parenteral lorazepam and as new medications are discovered and the ICU drug literature expands). Several next steps are proposed. First, given the present CDM does not include patient-level, order-specific, or case-specific temporal features, future research methodology focused on nuances of medication orders in the context of a specific patient and medication regimen is warranted. This research should focus on how combinations of medications may prompt increased scrutiny or medication order modification by ICU clinicians and would be intended to link individual patient encounters with interpretation of their drug therapy via this CDM. Indeed, pattern-recognition-based clinical reasoning models have been proposed in the pedagogy of medical education, and already the concept of AI-assisted pattern recognition for diagnosis is being investigated.^58^^,^^59^ Just as experience with a constellation of patient signs and symptoms can lead to rapid recognition of an ICU clinical diagnosis, medication use also has certain patterns.^59^^,^^60^ When an ICU clinician reviews the medication profile, each individual medication order is evaluated in a broader clinical context with the other medication orders. For example, a patient with one active vasopressor order likely registers very differently to the clinician than a patient requiring 3 vasopressors. Thus, in the context of comprehensive medication management, clinicians become increasingly attuned to “the realm of the unusual” for any medication regimen. Encoding medical expertise with some degree of level of evidence or strength of evidence, as is seen in clinical guidelines, could serve as a useful future addition to add further nuance to AI algorithms. Given that different use cases (eg, workload allocation, ADE prediction) likely require different datapoints, it is likely that “one size does not fit all” and different iterations of features may be needed, although these iterations require separate methodologies than used here. Finally, the list developed, while validated and studied as highly relevant to ICU patient outcomes, is not comprehensive. Linking a comprehensive feature list to existing databases like RxNorm or OMOP are important future investigations that will require governance and maintenance infrastructure.

## Conclusion

A modified Delphi process was applied to develop an initial standardized ontology of ICU medications for future use in AI research. This work represents a first step towards developing a CDM, and future research may focus on evaluation on validation and relevance of these features for making clinically relevant predictions in the ICU.

## Supplementary Material

ooae033_Supplementary_Data

## Data Availability

The data underlying this article are available in GitHub, under ICURx at: https://www.icurxforai.com/.
