# Supplementary material for: A common data model for the standardization of intensive care unit medication features
Source: JAMIA Open. 2024 May 2;7(2):ooae033. doi: 10.1093/jamiaopen/ooae033 (PMC11064096; doi:10.1093/jamiaopen/ooae033)
Supplement: ooae033_Supplementary_Data [file ooae033_supplementary_data.docx]

**Appendix I**

**Table 1. Respondent Votes for Round 2 Survey Questions**

| What is the level of priority for inclusion of this medication feature into a Common Data Model for ICU Medications? | | | | |
| --- | --- | --- | --- | --- |
|  | High Priority | Low Priority | Do not Include | Comment |
| Drug-Strength-Formulation (e.g., acyclovir 200mg capsule) | 4 | 4 | 0 | 0 |
| Drug Name (e.g., acyclovir) | 8 | 0 | 0 | 0 |
| Pharmaceutical class (e.g., antiviral agent) | 7 | 1 | 0 | 0 |
| Pharmaceutical subclass (e.g., DNA polymerase inhibitor) | 5 | 3 | 0 | 0 |
| Dose (e.g., 200) | 6 | 2 | 0 | 0 |
| Dose units (e.g., mg) | 5 | 3 | 0 | 0 |
| Volume, if intravenous (e.g., 100) | 3 | 4 | 1 | 0 |
| Concentration (e.g., 1mg/1mL) | 4 | 3 | 1 | 0 |
| Route (e.g., oral, intravenous) | 8 | 0 | 0 | 0 |
| IV subcategory (e.g., bolus vs. continuous infusion) | 7 | 1 | 0 | 0 |
| Bolus sub-category (e.g., IV push vs IV piggyback) | 4 | 3 | 1 | 0 |
| Oral sub-category (e.g., capsule, suspension) | 2 | 5 | 0 | 1 |
| Included in MRC-ICU | 6 | 1 | 0 | 1 |
| MRC-ICU Weight (i.e., 1,2,3) | 6 | 1 | 0 | 1 |
| Fixed route (i.e., only comes as oral agent) | 3 | 4 | 1 | 0 |
| Route escalation (e.g., IV instead of PO for same agent) | 6 | 2 | 0 | 0 |
| Therapeutic drug monitoring availability | 8 | 0 | 0 | 0 |
| Relative importance of Therapeutic Drug Monitoring in the ICU | 7 | 1 | 0 | 0 |
| Cytochrome P450 Enzyme | 6 | 1 | 1 | 0 |
| Pharmacogenomics | 3 | 4 | 1 | 0 |
| Dosing alterations are present due to critical illness | 7 | 0 | 0 | 1 |
| Drug is indicative critical illness | 7 | 0 | 0 | 1 |
| Weight-based dosing is used | 7 | 1 | 0 | 0 |
| Maximum daily dose | 5 | 2 | 1 | 0 |
| Maximum daily dose adjusted by levels of creatinine clearance | 5 | 2 | 1 | 0 |
| Renal dose adjustment | 6 | 2 | 0 | 0 |
| Hepatic dose adjustment | 6 | 2 | 0 | 0 |
| Contraindication in renal failure | 6 | 2 | 0 | 0 |
| Contraindication in hepatic failure | 6 | 2 | 0 | 0 |
| Used for prophylaxis of disease | 4 | 4 | 0 | 0 |
| ISMP High Alert Drug List^1^ | 3 | 4 | 1 | 0 |
| ISMP Confused Drug List^2^ | 1 | 6 | 1 | 0 |
| Beers Criteria^3^ | 1 | 6 | 1 | 0 |
| Continuous Infusion Starting Rate | 5 | 2 | 1 | 0 |
| Continuous Infusion Maximum Rate | 5 | 2 | 1 | 0 |
| Continuous Infusion units | 5 | 3 | 0 | 0 |
| What is the level of priority for inclusion of this medication monitoring feature into a Common Data Model for ICU Medications? | | | | |
| Creatinine Clearance | 7 | 1 | 0 | 0 |
| Serum creatinine | 6 | 2 | 0 | 0 |
| Urine output | 6 | 2 | 0 | 0 |
| Dialysis | 8 | 0 | 0 | 0 |
| Sodium | 5 | 3 | 0 | 0 |
| Potassium | 4 | 4 | 0 | 0 |
| Chloride | 3 | 5 | 0 | 0 |
| Bicarbonate | 5 | 3 | 0 | 0 |
| Blood urea nitrogen | 4 | 4 | 0 | 0 |
| Glucose | 6 | 2 | 0 | 0 |
| Magnesium | 6 | 2 | 0 | 0 |
| Phosphate | 5 | 3 | 0 | 0 |
| Liver function tests | 6 | 2 | 0 | 0 |
| Creatinine kinase | 5 | 3 | 0 | 0 |
| Cultures/Sensitivities | 8 | 0 | 0 | 0 |
| activated partial thromboplastin time (APTT) | 7 | 1 | 0 | 0 |
| Anti-Xa levels | 8 | 0 | 0 | 0 |
| Prothrombin time/international normalized ratio | 7 | 1 | 0 | 0 |
| Hemoglobin | 6 | 2 | 0 | 0 |
| Hematocrit | 4 | 4 | 0 | 0 |
| Platelets | 6 | 2 | 0 | 0 |
| White blood cell count | 6 | 2 | 0 | 0 |
| Patient weight | 7 | 1 | 0 | 0 |
| QTc Interval | 7 | 0 | 0 | 0 |
| Serum Osmolality | 4 | 4 | 0 | 0 |
| Ammonia | 4 | 4 | 0 | 0 |
| Absolute neutrophil count | 5 | 3 | 0 | 0 |
| Train of Four | 6 | 1 | 0 | 0 |
| Critical Care Pain Observation Tool or other Pain Score | 8 | 0 | 0 | 0 |
| Richmond Agitation and Sedation Scale (RASS) or other agitation/sedation score | 8 | 0 | 0 | 0 |
| Confusion Assessment Method for the Intensive Care Unit (CAM-ICU) | 7 | 1 | 0 | 0 |
| Mean arterial pressure | 8 | 0 | 0 | 0 |
| Heart Rate | 8 | 0 | 0 | 0 |
| Systolic blood pressure | 6 | 1 | 0 | 0 |
| Respiratory rate | 7 | 1 | 0 | 0 |
| 1. <https://www>.ismp.org/sites/default/files/attachments/2018-08/highAlert2018-Acute-Final.pdf. Accessed 11/17/22. 2. <https://www>.ismp.org/recommendations/confused-drug-names-list. Accessed 11/17/22. 3. By the 2019 American Geriatrics Society Beers Criteria® Update Expert Panel. American Geriatrics Society 2019 Updated AGS Beers Criteria® for Potentially Inappropriate Medication Use in Older Adults. J Am Geriatr Soc. 2019 Apr;67(4):674-694. | | | | |
| IV (intravenous); PO (oral); ICU (intensive care unit); (ISMP) Institute for Safe Medication Practices | | | | |

**Table 2. Round 4 Survey Voting Results**

| Feature | Core List for Most AI in the ICU | Core List for AI to Optimize Medication Use | Expanded List for AI to Optimize Medication Use |
| --- | --- | --- | --- |
| Drug Product [e.g., Drug-Strength-Formulation, acyclovir 200mg capsule] | 5 | 2 | 0 |
| Drug Name (e.g., acyclovir) | 8 | 0 | 0 |
| Therapeutic Category | 6 | 0 | 1 |
| Pharmacologic Class | 6 | 0 | 1 |
| Formulary Key Drug Types | 3 | 2 | 1 |
| Formulation Strength | 7 | 2 | 1 |
| Formulation Strength Conversion (e.g., for beta-blockers, metoprolol equivalents) | 3 | 4 | 0 |
| Strength Units (e.g. mg) | 7 | 1 | 0 |
| Intravenous Volume | 3 | 5 | 0 |
| Concentration | 3 | 5 | 0 |
| Concentration Units | 2 | 5 | 0 |
| Route | 8 | 0 | 0 |
| Intravenous route subcategory (e.g., bolus vs. continuous infusion) | 7 | 1 | 0 |
| Bolus sub-category (e.g., intravenous push vs intravenous piggyback) | 4 | 3 | 1 |
| Oral sub-category (e.g., capsule, suspension) | 3 | 5 | 0 |
| Fixed route (i.e., only comes as oral agent) | 3 | 2 | 2 |
| Route escalation (e.g., intravenous instead of oral for same agent) | 5 | 2 | 0 |
| **Feature** |  |  |  |
| **Laboratory, vital sign, and other monitoring parameters** | | | |
| Creatine clearance | 8 | 0 | 0 |
| Serum creatinine | 7 | 1 | 0 |
| Urine output | 7 | 1 | 0 |
| Liver function tests | 7 | 1 | 0 |
| Magnesium | 4 | 4 | 0 |
| Sodium | 5 | 3 | 0 |
| Dialysis | 8 | 0 | 0 |
| Potassium | 5 | 3 | 0 |
| Chloride | 4 | 4 | 0 |
| Bicarbonate | 5 | 3 | 0 |
| Blood urea nitrogen | 4 | 4 | 0 |
| Glucose | 6 | 2 | 0 |
| Phosphate | 3 | 4 | 1 |
| Creatinine Kinase | 4 | 3 | 1 |
| Cultures/Sensitivities | 8 | 0 | 0 |
| activated partial thromboplastin time (APTT) | 5 | 2 | 1 |
| Anti-Xa levels | 5 | 2 | 1 |
| Prothrombin time/international normalized ratio | 6 | 1 | 1 |
| Hemoglobin | 4 | 4 | 0 |
| Hematocrit | 3 | 5 | 0 |
| Platelets | 5 | 3 | 0 |
| White blood cell count | 6 | 2 | 0 |
| Patient weight | 8 | 0 | 0 |
| QTc Interval | 5 | 2 | 1 |
| Serum osmolality | 2 | 5 | 1 |
| Ammonia | 3 | 4 | 1 |
| Absolute neutrophil count | 5 | 2 | 1 |
| Train of Four | 4 | 4 | 0 |
| Critical Care Pain Observation Tool or other Pain Score | 8 | 0 | 0 |
| Richmond Agitation and Sedation Scale (RASS) or other agitation/sedation score | 8 | 0 | 0 |
| Confusion Assessment Method for the Intensive Care Unit (CAM-ICU) | 7 | 1 | 0 |
| Mean arterial pressure | 8 | 0 | 0 |
| Heart Rate | 7 | 1 | 0 |
| Systolic blood pressure | 6 | 2 | 0 |
| Respiratory rate | 6 | 2 | 0 |
| Bowel movements | 6 | 2 | 0 |
| Triglycerides, Lipid panel | 4 | 4 | 0 |
| Temperature | 6 | 2 | 0 |
| **Feature** |  |  |  |
| Included in MRC-ICU | 7 | 1 | 0 |
| MRC-ICU Weight (i.e., 1,2,3) | 6 | 1 | 0 |
| Route Escalation | 6 | 2 | 0 |
| Weight-based dosing is used | 8 | 0 | 0 |
| Maximum daily dose | 6 | 2 | 0 |
| Maximum Daily Dose (absolute value) | 6 | 1 | 1 |
| Maximum daily dose adjusted by levels of creatine clearance | 7 | 1 | 0 |
| Renal dose adjustment | 8 | 0 | 0 |
| Hepatic dose adjustment | 7 | 1 | 0 |
| Contraindication in renal failure | 8 | 0 | 0 |
| Contraindication in hepatic failure | 8 | 0 | 0 |
| Used for prophylaxis of disease | 4 | 4 | 0 |
| Dose in prophylaxis settings | 3 | 5 | 0 |
| Patient acuity/Drug is indicative critical illness | 7 | 1 | 0 |
| Critical illness based dosing/ Dosing alterations are present due to critical illness | 7 | 1 | 0 |
| Institute for Safe Medication Practices (ISMP) High Alert | 5 | 3 | 0 |
| ISMP Confused Drug List | 4 | 3 | 1 |
| Beers Criteria | 2 | 6 | 0 |
| Continuous Infusion Starting Rate | 4 | 3 | 0 |
| Continuous Infusion Maximum Rate | 6 | 2 | 0 |
| Continuous Infusion units | 4 | 4 | 0 |
| Therapeutic Drug Monitoring | 8 | 0 | 0 |
| Parameter based drug dosing (e.g., glucose for insulin) | 8 | 0 | 0 |
| Cytochrome P450 Enzyme Inhibitor, Inducer, Substrate | 6 | 2 | 0 |
| Pharmacogenomics | 2 | 6 | 0 |
| Drug is indicative of critical illness | 7 | 1 | 0 |
| Please review the case vignette. Then, consider the *features of a medication product* that would be most relevant/most important to clinical decision-making associated with comprehensive medication management of that patient. One of four categories can be selected: 1) Use is recommended for most AI/ICU applications/part of “core” list 2) Use is recommended for most AI/ICU applications related to optimization of medication therapy in the ICU/part of “core list” 3) Potential relevance in expanded medication list, mostly related to optimization of medication therapy in the ICU 4) Comment Note: This category does not include “sig specific” components like the frequency, titration parameters, etc. This will be a phase 2 experience. Case Vignette: A 36 year old male is admitted to the medical ICU for hypotension refractory to fluid resuscitation and hypoxia requiring invasive positive pressure ventilation. He is diagnosed with sepsis secondary to community acquired pneumonia. Chest X-ray reveals bilateral pulmonary infiltrates, and his most recent ABG is pH 7.3, PaO2 105, PaCO2 32, HCO3 21 on 100% FiO2. MAPs are currently ranging 59-66. His current medication profile is as follows: acetaminophen 325 mg tablet q4h prn temperature cefepime 2gm in 100mL NS q8h given IV as extended infusion over 2 hours cisatracurium as a titratable continuous infusion chlorhexidine 15mL oral swab BID dexamethasone 20mg tablet daily docusate 100mg capsule BID eye lubricant BID prn dry eyes famotidine 20mg tablet BID fentanyl 20mcg/mL in 100mL NS as a titratable continuous infusion fentanyl 100mcg q30min IV push prn per sedation guideline heparin 7,500 units sub-cutaneous injection q8h norepinephrine 12mg in 250mL D5W as a titratable continuous infusion propofol 1000mg in 100mL as a titratable continuous infusion D5W/0.45%NaCl with 20meq potassium chloride at 50mL/hour continuous infusion senna 187mg tablet BID vancomycin 1250mg in 250mL NS q8h vasopressin 40units/100mL D5W at 0.04 units/min | | | |
| IV (intravenous); PO (oral); ICU (intensive care unit); (ISMP) Institute for Safe Medication Practices | | | |

**Table 3.** Common Data Model for ICU Medications

| **Category 1: Drug Product Features** | | | | |
| --- | --- | --- | --- | --- |
| **Feature** | | **Definition** | **Response Values** | **Data Value** |
| Drug Product | | Complete information to uniquely identify a particular drug product including drug name, strength, units, and formulation | Name-Strength-Formulation  Example: “Aspirin 81mg tablet” | Alpha-numerical |
| Drug Name | | Drug name in Lexi-Comp compendium | Name  Example: “Aspirin” | Alpha-numerical |
| Therapeutic Category | | Classification based on therapeutic action in the body per AHFS Pharmacologic-Therapeutic Classification | Therapeutic Category Name  Example: “Cardiovascular Agents” | Alpha-numerical |
| Pharmacologic Class | | Name for a group of active moieties that share scientifically documented properties defined on the basis of any combination of three attributes of the active moiety: Mechanism of Action, Physiologic Effect, Chemical Structure per U.S. Food & Drug Administration | Pharmacologic Class Name  Example: “Antiarrhythmics” | Alpha-numerical |
| Formulary Key Drug Types | | Sub-classification of pharmacologic class based on Mechanism of Action, Physiologic Effect, Chemical Structure per U.S. Food & Drug Administration | Formulary Key Drug Types  Example: “Antiarrhythmics – Class IV” | Alpha-numerical |
| Formulation Strength | | Describes the amount of active ingredient in the substance | Numeric amount  Example: For aspirin, “81” | Continuous, numerical |
| Formulation Strength Conversion | | For distinct classes of drugs (e.g., beta-blockers), dose-conversion guidelines are available and were applied to provide approximate dose equivalency | 0: no, 1: yes | Categorical, coded |
| Converted Drug Name | | Name of drug that provides dose equivalency | Name  Example: “Aspirin” | Alpha-numerical |
| Converted Drug Strength | | Strength of drug that provides dose equivalency | Numeric amount  Example: For aspirin, “81” | Continuous, numerical |
| Strength Units | | Unit of measure for formulation strength | \| Units (U) \| 1 \| \| --- \| --- \| \| Milligrams per milliliter (mg/mL) \| 2 \| \| Percent (%) \| 3 \| \| Units per milliliter (U/mL) \| 4 \| \| Grams (g) \| 5 \| \| Grams per milliliter (g/mL) \| 6 \| \| Micrograms per milliliter (mcg/mL) \| 7 \| \| Micrograms (mcg) \| 8 \| \| Milligram/kilogram (mg/kg) \| 9 \| \| Milliliters (mL) \| 10 \| \| Milligrams (mg) \| 11 \| \| mL/kg \| 12 \|   Example: for milligram, “11” | Categorical, coded |
| Route | | Location at which the drug is introduced to the body | \| Intravenous \| 1 \| \| --- \| --- \| \| Topical \| 2 \| \| Subcutaneous \| 3 \| \| Transdermal \| 4 \| \| Intramuscular \| 5 \| \| Rectal \| 6 \| \| Eye drop \| 7 \| \| Inhalation \| 8 \| \| Oral \| 9 \| \| Enteral \| 10 \|   Example: “Subcutaneous” | Categorical, coded |
| Intravenous Volume | | For intravenous products, total volume in milliliters (mL) of the Drug Product to be administered to the patient | Numeric amount  Example: “100” | Continuous, numerical |
| Concentration | | Amount of drug in a given volume | Numeric amount  Example: “10” | Continuous, numerical |
| Concentration Units | | Unit of measure for the amount of drug per volume | \| Milligrams per milliliter (mg/mL) \| 1 \| \| --- \| --- \| \| Units per milliliter (U/mL) \| 2 \| \| Percent (%) \| 3 \| \| Millimoles per milliliter (mmol/mL) \| 4 \| \| Milliequivalents per milliliter (mEq/mL) \| 5 \|   Example: for mg/mL, “1” | Categorical, coded |
| Intravenous, sub-category | | Categorization of product formulation given as an intravenous bolus | \| IV Piggyback \| 1 \| \| --- \| --- \| \| IV Push \| 2 \| \| Continuous Infusion \| 3 \| \| Extended Infusion \| 4 \| \| Fluid Bolus \| 5 \| \| Not applicable \| 0 \| | Categorical, coded |
| Oral, sub-category | | Categorization of product formulation given orally | \| Capsule \| 1 \| \| --- \| --- \| \| Solution/Suspension \| 2 \| \| Powder/Granules \| 3 \| \| Tablet \| 4 \| \| Not applicable \| 0 \|   Example: for tablet, “4” | Categorical, coded |
| **Category 2: Clinical Features** | | | | |
| ***Dosing Information*** | | | | |
| **Feature** | **Definition** | | **Response Values** | **Data Value** |
| Fixed Route | This particular drug (per drug name) can only be given via one route (e.g., only oral formulations are available)  Note: a drug that is only given oral (but route of by mouth vs. via feeding tube would be counted as a single route available). | | \| Multiple routes available \| 1 \| \| --- \| --- \| \| Single route available \| 2 \|   Example: for multiple routes, “1” | Categorical, coded |
| Weight-based dosing | Categorizes medications based on dosing that is standard across weight ranges (e.g., aspirin 81mg for all cardiovascular risk prevention) vs. weight-based (e.g., vancomycin loading dose 25mg/kg) | | \| Weight-based dosing is available but not routinely used \| 1 \| \| --- \| --- \| \| Doses are calculated using the patient’s weight \| 2 \| \| A nomogram is used to calculate dose adjustments in patients with impaired renal function \| 3 \| \| Doses are standardized \| 4 \| \| Combinations of weight-based and nomograms are used \| 5 \|   Example: for weight-based dosing, “2” | Categorical, coded |
| Maximum Daily Dose Available | Yes, if a specified maximum dose is listed in the Dosing: Adult section in Lexicomp. | | \| Maximum doses are well defined \| 0 \| \| --- \| --- \| \| Maximum doses are less clearly defined or not applicable (e.g., differences exist between package insert & clinical practice) \| 1 \| \| Example: for no maximum dose, “0” \|  \| | Categorical, coded |
| Maximum Daily Dose Value | Based on highest daily dose in Dosing: Adult sections in Lexicomp.  For medications using weight-based dosing and an absolutely value was derived by using 100kg as the weight (e.g., gentamicin 5mg/kg 🡪 500mg as a ‘maximum’ dose for machine-reading purposes) | | Example: For acetaminophen, “4000” to denote maximum daily dose of 4000mg | Continuous, numeric |
| Maximum Daily Strength Units | Unit of measure for formulation strength | | Refer to Strength Units | Categorical, coded |
| Maximum daily dose for CrCl < 10 / hemodialysis | Based on institutional consensus guidelines | | Example: “500” for meropenem | Continuous, numeric |
| Maximum daily dose for CrCl = 10-29 |  |  | Example: “1000” for meropenem |  |
| Maximum daily dose for CrCl = 30-49 |  |  | Example: “2000” for meropenem |  |
| Maximum daily dose for CrCl < 10 / hemodialysis AND critically ill | Based on institutional consensus guidelines for when indication of the medication is directly associated with need for ICU | | Example: “1000” for meropenem for *Pseudomonas* pneumonia with high MIC | Continuous, numeric |
| Maximum daily dose for CrCl 10-29 AND critically ill |  |  | Example: “2000” for meropenem for *Pseudomonas* pneumonia with high MIC |  |
| Maximum daily dose for CrCl 30-49 AND critically ill |  |  | Example: “4000” for meropenem for *Pseudomonas* pneumonia with high MIC |  |
| Maximum daily dose for CrCl >50 AND critically ill |  |  | Example: “6000” for meropenem for *Pseudomonas* pneumonia with high MIC |  |
| Maximum daily dose for CrCl <10 hemodialysis AND has cystic fibrosis* | Based on institutional consensus guidelines | | Example: “10” for tobramycin | Continuous, numeric |
| Maximum daily dose for CrCl 10-29 AND has cystic fibrosis* |  |  |  |  |
| Maximum daily dose for CrCl 30-49 AND has cystic fibrosis* |  |  |  |  |
| Maximum daily dose for CrCl >50 AND has cystic fibrosis* |  |  |  |  |
| Renal Dose Adjustment | Based on Dosing: Altered Kidney Function: Adult section in Lexicomp | | 0: no, 1: yes | Categorical, coded |
| Hepatic dose adjustment | Based on Dosing: Hepatic Impairment in Lexicomp | |  |  |
| Contraindicated in renal failure | Based on Dosing: Altered Kidney Function in Lexicomp | |  |  |
| Contraindicated in hepatic failure | Based on Dosing: Hepatic Impairment in Lexicomp | |  |  |
| Role in prophylaxis | This medication can be used for prophylaxis and has dosing specific to prophylaxis vs. treatment of an indication (e.g., prevention of deep venous thrombosis with enoxaparin) | |  |  |
| Maximum prophylactic dose | Maximum dose is available for indications of prophylaxis | | Numeric dose | Categorical, coded |
| Maximum prophylactic dose units | Unit of measure for formulation strength | | Refer to Formulation strength | Categorical, coded |
| Continuous Infusion Starting Rate | For intravenous continuous infusions, the rate of administration at which the drug is typically initiated | | Numeric amount  Example: “5” | Continuous, numeric |
| Continuous Infusion Maximum Rate | For intravenous continuous infusions, the maximum rate of administration | | Numeric amount  Example: “15” | Continuous, numeric |
| Units for continuous infusion | Unit of measure for continuous infusion | | \| Units per minute (U/min) \| 1 \| \| --- \| --- \| \| Units per kilogram per minute (U/kg/min) \| 2 \| \| Units per hour (U/h) \| 3 \| \| Units per kilogram per hour (u/kg/h) \| 4 \| \| Milligrams per minute (mg/min) \| 5 \| \| Milligrams per kilogram per minute (mg/min) \| 6 \| \| Milligrams per hour (mg/h) \| 7 \| \| Milligrams per kilogram per hour (mg/kg/h) \| 8 \| \| Micrograms per minute (mcg/min) \| 9 \| \| Micrograms per kilogram per minute (mcg/kg/min) \| 10 \|   Example: for mg/min, “5” | Continuous, numeric |
| ***Laboratory Monitoring*** | | | | |
| **Feature** | **Definition** | | **Response Values** | **Data Value** |
| Serum Creatinine | All monitoring parameters were marked as “yes” if the parameter would be either monitored on a regular (at least weekly) basis while the patient is receiving the medication or if a certain baseline value of the parameter may cause a clinician to not utilize a specific medication or alter the starting dose. | | 0: no, 1: yes | Categorical, coded |
| Aspartate Aminotransferase |  |  |  |  |
| Alanine Transaminase |  |  |  |  |
| Magnesium |  |  |  |  |
| Sodium |  |  |  |  |
| Potassium |  |  |  |  |
| Phosphorous |  |  |  |  |
| Activated partial thromboplastin time |  |  |  |  |
| Anti-Xa |  |  |  |  |
| Microbiology results (cultures, sensitivity, rapid results) |  |  |  |  |
| Hemoglobin |  |  |  |  |
| Platelets |  |  |  |  |
| Absolute neutrophil count |  |  |  |  |
| Creatinine Kinase |  |  |  |  |
| White blood cell count |  |  |  |  |
| Serum Osmolality |  |  |  |  |
| Albumin |  |  |  |  |
| International normalized ratio (INR) |  |  |  |  |
| Ammonia |  |  |  |  |
| Glucose |  |  |  |  |
| Triglycerides, lipid panel |  |  |  |  |
| ***Vital Sign and other ICU Monitoring*** | | | | |
| **Feature** | **Definition** | | **Response Values** | **Data Value** |
| Creatinine Clearance | All monitoring parameters were marked as “yes” if the parameter would be either monitored on a regular (at least weekly) basis while the patient is receiving the medication or if a certain baseline value of the parameter may cause a clinician to not utilize a specific medication or alter the starting dose. | | 0: no, 1: yes | Categorical, coded |
| Urine output |  |  |  |  |
| Bowel movements |  |  |  |  |
| Dialysis |  |  |  |  |
| Patient Weight |  |  |  |  |
| QTc Interval |  |  |  |  |
| Train of Four |  |  |  |  |
| Pain Scale |  |  |  |  |
| Sedation scale |  |  |  |  |
| Delirium (CAM-ICU) |  |  |  |  |
| Mean arterial pressure |  |  |  |  |
| Systolic blood pressure |  |  |  |  |
| Heart Rate |  |  |  |  |
| Systolic blood pressure |  |  |  |  |
| Respiratory rate |  |  |  |  |
| Temperature |  |  |  |  |
| ***Clinical-Decision Making*** | | | | |
| **Feature** | **Definition** | | **Response Values** | **Data Value** |
| MRC-ICU Presence | Classification of medication based on inclusion in the MRC-ICU Scoring Tool | | 0: no, 1: yes  Example: For vancomycin, “1” | Categorical, coded |
| MRC-ICU Weight | Classification of weight per the MRC-ICU Scoring Tool | | 1, 2, 3  Example: For vancomycin, “3” | Categorical, numerical |
| Route Escalation | Different routes of drugs may have different efficacy or be used in conditions of critical illness to ensure optimal activity (e.g., giving intravenous antibiotics instead of oral antibiotics). | | \| Single route available \| 1 \| \| --- \| --- \| \| Multiple routes are available and IV is therapeutically superior (e.g., used for improved efficacy in patient-specific scenarios, including concern for reduced absorption via the gut) to all other routes \| 2 \| \| Multiple routes are available but they are all equivalent \| 3 \| | Categorical, coded |
| Patient Acuity | This feature indicates where the medication is most likely to be used as an indicator of patient acuity based on two independently adjudicated clinician perspectives | | \| Theedicationn can be used in an inpatient and outpatient setting and the dose is the same in both settings \| 1 \| \| --- \| --- \| \| The medication is only used in the hospital setting in both critically ill and non-critically ill \| 2 \| \| The medication is only used in patients who are critically ill \| 3 \| \| The medication is used in an inpatient and outpatient setting, but the dose will differ between both settings \| 4 \| | Categorical, coded |
| Critical Illness Based Dosing | Drugs were marked accordingly if the dosing would be different in a patient who was critically ill compared to a patient receiving the same medication but was not critically ill. Alternatively, some indications may have different doses and certain indications would qualify the patient as critically ill. Alternatively, dosing may be different for separate indications but none of the indications would necessarily qualify a patient as critically ill. Finally, some drugs may have flat doses with no changes between indications. | | \| Same indications have a different “ICU dose” compared to a “floor dose” \| 0 \| \| --- \| --- \| \| Specific indications (associated with critical illness) have different doses \| 1 \| \| Dosing may be different for different indications, but no indication is a “critically ill” indication \| 2 \| \| All doses are flat and all indications have the same dosing \| 3 \| | Categorical, coded |
| Drug Indicates Critical Illness | Drugs were marked as “always” if in seeing that a patient was receiving a certain indication, a clinician would know that the patient was critically ill, “maybe” if the indication and/or dosing would potentially infer that the patient was critically ill, or “never” if nothing about the medication, dosing, or indication is specific to critical illness. | | \| Always \| 0 \| \| --- \| --- \| \| Maybe, depending on indication and/or dose \| 1 \| \| Never \| 2 \| | Categorical, coded |
| Adverse Events | Medications were marked according to estimated percentage of patients experiencing adverse events based on two independently adjudicated clinician perspectives | | \| Expected adverse events <10% \| 1 \| \| --- \| --- \| \| Expected adverse events 10-49% \| 2 \| \| Expected adverse events 50-100% \| 3 \| | Categorical, coded |
| Realm of the Unusual | Medications were marked according to likelihood of a practitioner to use this drug in an ICU setting during the course of a year based on two independently adjudicated clinician perspectives | | \| Very unusual (<1 times/year) \| 1 \| \| --- \| --- \| \| Moderately unusual (<5 times/year) \| 2 \| \| Somewhat unusual (<20 times/year) \| 3 \| \| Common (At least weekly use) \| 4 \| \| Very common (Daily use) \| 5 \| | Categorical, coded |
| ISMP High Alert Status | This medication is present on the ISMP High Alert Medication List. | | 0: no, 1: yes | Categorical, coded |
| ISMP Confused Drug Names | This medication is present on the ISMP Confused Drug Names list. | |  |  |
| Beers Criteria | This medication is present on the Beers Criteria list. | |  |  |
| Beers Criteria Strong Recommendation | This medication is categorized as a strong recommendation on the Beers Criteria list. | |  |  |
| Therapeutic Drug Monitoring | Therapeutic drug monitoring was defined as “always relevant” if it would be standard of care to monitor on every admission, “potentially relevant in ICU” if it would be reasonable to monitor in some patients if relevant but would not be required for all patients, “not relevant” if the drug would not be monitored on the inpatient setting, or unavailable if no therapeutic drug monitoring exists for this medication. Of note, TDM means that serum drug levels can be routinely obtained and are clinically meaningful to interpret and confirm medication dose. | | \| Always relevant \| 1 \| \| --- \| --- \| \| Potentially relevant \| 2 \| \| Not relevant \| 3 \| \| Unavailable \| 4 \|   Example: for vancomycin, “1” | Categorical, coded |
| Parameter based dosing | Drug doses are guided specifically on monitoring parameters including biomarkers or vital signs (e.g., insulin is based on glucose) | | 0: no, 1: yes | Categorical, coded |
| CYP3A4 Interaction | The drug interacts with CYP3A4 in the following way. | | \| Major Inducer \| 1 \| \| --- \| --- \| \| Major Inhibitor \| 2 \| \| Major Substrate \| 3 \| \| No or minor interaction \| 4 \| | Categorical, coded |
| CYP2D6 Interaction | The drug interacts with CYP2D6 in the following way. | |  |  |
| CYP1A2 Interaction | The drug interacts with CYP1A2 in the following way. | |  |  |
| CYP2C9 Interaction | The drug interacts with CYP2C9 in the following way. | |  |  |
| CYP2C19 Interaction | The drug interacts with CYP2C19 in the following way. | |  |  |
| P-glycoprotein Interaction | The drug interacts with P-glycoprotein in the following way. | |  |  |
| Pharmacogenomics | This drug is present on the FDA Pharmacogenomics listing | | 0: no, 1: yes | Categorical, coded |
| Broad versus Narrow Spectrum | Antimicrobials were marked as broad versus narrow spectrum of activity based on two independently adjudicated clinician perspectives | | \| Broad Spectrum Agent \| 1 \| \| --- \| --- \| \| Narrow Spectrum Agent \| 2 \| \| Not Applicable \| 3 \| | Categorical, coded |
| Vancomycin-Resistant *Enterococcus spp.* | Antimicrobials were marked by willingness of a clinician to use them for a resistant infection based on type of infection and severity of illness | | \| Would use for all sources of infection and in critically ill patients \| 1 \| \| --- \| --- \| \| Would use for some sources of infection and in critically ill patients \| 2 \| \| Would use for some sources of infection but not in critically ill patients \| 3 \| \| Would not use for this infection \| 4 \| \| Not Applicable \| 5 \| | Categorical, coded |
| Methicillin-Resistant *Staphylococcus Aureus* |  |  |  |  |
| *Pseudomonas aeruginosa* |  |  |  |  |
| Extended Spectrum Beta Lactamase |  |  |  |  |
| Treatment Failure | Medications were marked according to estimated percentage of treatment failure based on two independently adjudicated clinician perspectives | | \| Expected treatment failure <10% \| 1 \| \| --- \| --- \| \| Expected treatment failure 10-49% \| 2 \| \| Expected treatment failure 50-100% \| 3 \| | Categorical, coded |
| IV (intravenous); PO (oral); ICU (intensive care unit); (ISMP) Institute for Safe Medication Practices; CrCl (creatinine clearance) | | | | |

*cystic fibrosis dosing was only reported for aminoglycosides
